# Assessment of liver stiffness and steatosis in patients with intestinal failure: a cross-sectional study

**DOI:** 10.3389/fnut.2025.1718310

**Published:** 2025-12-11

**Authors:** Ena Muhic, Rahim Mohammad Naimi, Christopher Filtenborg Brandt, Thomas Lund Andersen, Helle Hjorth Johannesen, Palle Bekker Jeppesen

**Affiliations:** 1 Section of Intestinal Failure, Department of Digestive Diseases, Transplantation and General Surgery, Copenhagen University Hospital - Rigshospitalet, Copenhagen, Denmark; 2 Department of Clinical Physiology and Nuclear Medicine, Copenhagen University Hospital - Rigshospitalet, Copenhagen, Denmark; 3 Department of Clinical Medicine, Copenhagen University, Copenhagen, Denmark

**Keywords:** parenteral nutrition, intestinal failure, home parenteral support, intestinal failure-associated liver disease, short bowel syndrome, steatosis, liver stiffness

## Abstract

**Introduction:**

Intestinal failure-associated liver disease is a potentially life-threatening complication in patients with intestinal failure (IF) receiving home parenteral support (HPS). The aim of this study was to evaluate liver stiffness and steatosis non-invasively in adult patients with IF receiving long-term HPS.

**Methods:**

Patients with IF on a stable HPS prescription (≥4 days/week for ≥10 weeks) were included in this cross-sectional study. Liver stiffness measurements (LSMs) were assessed by transient elastography, and steatosis by MRI-proton density fat fraction (MRI-PDFF). Blood samples were analyzed for liver function. Data were compared to sex- and age-matched controls.

**Results:**

A total of 33 patients (median age, 57 years; BMI, 23 kg/m^2^) and 14 controls (median age, 58 years; BMI, 24 kg/m^2^) were examined. Patients had higher LSMs compared to controls (5.6 kPa vs. 4.3 kPa; *p* = 0.0079). Elevated LSMs (>7 kPa) were seen in 37% of patients vs. 0% in controls (*p* = 0.0085). LSMs were associated with HPS energy (*p* = 0.026) and lipid content (*p* = 0.029). Median MRI-PDFF was 1.8% in patients and 1.5% in controls (*p* = 0.43). Nineteen percent of patients exhibited elevated MRI-PDFF (≥8%) vs. 7% in controls (*p* = 0.41). Alkaline phosphatase and gamma-glutamyltransferase levels >1.5x the ULN were observed in 21 and 45% of patients, respectively. Overall, these results indicate that in this adult IF population, elevated LSMs and a cholestatic pattern in liver enzymes were more prevalent than steatosis as assessed by MRI-PDFF. Patients at risk of increased liver stiffness appeared to have severe IF as indicated by high HPS energy and lipid requirements.

## 1 Introduction

Patients with chronic intestinal failure (IF) are potentially dependent on life-long home parenteral support (HPS). Although lifesaving, IF and HPS may cause intestinal failure-associated liver disease (IFALD), which in centers of experience is reported to be the cause of 4–6% of all HPS-related deaths in adults (1–8). Depending on the definition, IFALD is characterized by persistent liver dysfunction, which can manifest as abnormal liver biochemistry, steatosis, cholestasis, fibrosis, and potentially progress to cirrhosis and end-stage liver failure (9–12). Currently, no uniform, standardized diagnostic criteria for IFALD exist, resulting in large variability in reported prevalence and incidence rates, ranging from 0 to 50%, with the highest rates observed in children (9, 11, 13). The European Society for Clinical Nutrition and Metabolism recently recommended diagnosing IFALD by *“the presence of abnormal liver function tests and/or evidence of radiological and/or histological liver abnormalities occurring in an individual with IF, and occurring in the absence of another primary parenchymal liver pathology* (e.g.*, viral or autoimmune hepatitis*), *other hepatotoxic factors* (e.g.*, alcohol/medication*) *or biliary obstruction”* (14). Still, liver histology is not mandatory for the diagnosis given the invasive nature and risk of the procedure (11, 14). Therefore, non-invasive tests may become increasingly important in the future as supportive tools for evaluating liver injury in patients with IF.

The etiology of IFALD is multifactorial, involving both HPS and patient-related risk factors (9, 10, 12, 15, 16). The HPS-associated risk factors include its composition, continuous infusions, overload of glucose, lipids, and other nutrients as well as deficiencies (9, 12, 17, 18). Glucose overload can lead to increased insulin secretion, which may induce lipogenesis and hepatic lipid accumulation (9, 10, 12, 17). Furthermore, high doses of lipids can exceed the capacity of the liver to metabolize phospholipids and fatty acids and contribute to steatosis (9, 10, 17, 18). Lipid emulsions derived from soybean oils have been specifically associated with liver injury due to their high content of phytosterols and pro-inflammatory omega-6 polyunsaturated fatty acids (9, 10, 12, 19). On the other hand, deficiencies in nutrients such as essential fatty acids, choline, carnitine, and vitamins C and E may also contribute to steatosis and liver injury by interfering with the lipid metabolism (10, 12, 16).

Patient-related risk factors associated with IFALD have been hypothesized to include a short length of remaining small bowel, particularly < 100 cm in adults (12, 20, 21), a lack of enteral nutrition, intestinal dysbiosis, bacterial translocation of hepatotoxic and inflammatory compounds into the portal circulation, repeated episodes of sepsis, and disturbances in the normal enterohepatic bile salt circulation (9, 10, 16, 18).

Although there have been significant advancements in the clinical management of patients with IF and HPS over recent decades, IFALD is perhaps best described as an umbrella term for a complex, multifaceted, and potentially fatal liver condition with underlying mechanisms that remain poorly understood and difficult to elucidate. This study aims to assess and describe markers of liver injury in patients with IF using non-invasive methods with a focus on liver stiffness, steatosis, and liver function tests.

## 2 Materials and methods

### 2.1 Study design and population

This study is a sub-study of a larger multicenter cross-sectional observational study (Clinical Trial Number: NCT05011370). Adult patients with chronic IF, aged 18 to 80 years, were recruited in the period from November 2022 to September 2023, from the intestinal failure unit at Copenhagen University Hospital, Rigshospitalet. The study size was based on the number of eligible patients during the study period. To be eligible for participation, patients were required to be on a stable HPS program of at least 4 days per week, for 10 weeks or longer. A stable HPS program was defined as receiving glucose (up to 35 kcal/kg/day), amino acids, vitamin B12 and folic acid in each infusion for at least 6 weeks prior to screening, and with no change in average energy provision during this period. The HPS prescription could either contain no lipids or include lipids at a maximum of 1.0 g/kg/day. A minimum 10-week period of stable HPS program was chosen to ensure that patients were in a metabolically stable phase representative of chronic IF, as shorter durations might include patients in transient, post-surgical, or otherwise unstable phases that could affect outcomes. Patients who received steatogenic or hepatotoxic medication, had an active infection, malignancy, history of organ transplant, recent surgery, cystic fibrosis, pregnancy, non-alcoholic steatohepatitis, or body mass index (BMI) < 15 or > 35 kg/m^2^, were excluded.

A group of age- and sex-matched volunteers without clinically significant diseases were included to serve as a healthy control group. The exclusion criteria, applied to the patient group, were also applied to the control group. The control group was required to fast for at least 2 h prior to all examinations, while the patients were assessed under their habitual conditions; thus, not necessarily fasting, and having HPS infused as habitually prescribed.

The study was conducted as a single day visit in which clinical data collection, blood sampling, and imaging assessment were performed. Clinical data included a physical examination of height, weight, and vital signs, as well as collection of information related to medical history and HPS. The latter was obtained by interview and a review of medical records and HPS prescriptions. The energy conversion factors of 17.6, 23.6, and 39.1 kJ/g for HPS carbohydrate, protein, and fat amounts, respectively, were used to convert HPS energy content (22).

### 2.2 Non-invasive imaging modalities

Imaging assessments were performed on the same day, starting with FibroScan® and followed by magnetic resonance imaging (MRI)-derived proton density fat fraction (PDFF) examination within two hours.

The non-invasive device FibroScan® 502 Touch (Echosens, Paris, France), which employs transient elastography (TE) technology, was used to assess liver stiffness and steatosis (23–25). The TE examination was performed with the patients and controls in a supine position, with the right arm raised above the head in maximal abduction. The probe was placed in the intercostal space corresponding to the right lobe of the liver, and an appropriate probe size (M or XL) was selected based on the recommendations from the automatic probe selection tool on the FibroScan®. The liver stiffness measurements (LSMs) are expressed in kilopascal (kPa), and values ≥ 7 were considered elevated (25, 26). Simultaneously with evaluating liver stiffness, steatosis was also assessed by the FibroScan® as the controlled attenuation parameter (CAP), expressed in dB/m. CAP values above 248 dB/m were considered elevated, indicating steatosis (27).

LSMs and CAP values were calculated as the median of at least 10 valid measurements and considered reliable if the success rate was ≥ 60% and interquartile range/median ratio was ≤ 30% (23). The examination was classified as a technical failure if a valid examination was not obtained, or if the measurements were considered unreliable. All examinations were performed by an experienced operator.

A further measurement of hepatic steatosis was performed by multi-echo MRI-PDFF with T2* correction (28). The MRI-PDFF was expressed as a percentage and calculated as the mean of 3 representative regions of interest (ROI), which included both the right and left liver lobes. Each ROI had a minimum radius of ~ 2 cm, avoiding large vessels, dilated bile ducts, and image artifacts and was placed by an experienced radiologist (28, 29). All MRIs were performed on a Biograph mMR integrated PET/MRI scanner (Siemens Healthtineers, Erlangen, Germany). MRI-PDFF values ≥ 8% were deemed abnormal.

The multi-echo MRI-PDFF with T2* correction was calculated using 6 echoes with echo times of 1.05 ms., 2.46 ms., 3.69 ms., 4.92 ms., 6.15 ms., and 7.38 ms. with a repetition time of 9.00 ms. The scan range covered a 450 mm field of view, and 64 slices with a slice thickness of 3.5 mm captured in a single breath hold and was distortion corrected. Multi-echo data was subsequently reconstructed by an iterative decomposition of water and fat with echo asymmetry and least-squares estimation simultaneously estimating fat fraction and T2*.

### 2.3 Laboratory parameters

Liver function tests included alkaline phosphatase (ALP), alanine aminotransferase (ALT), aspartate aminotransferase (AST), total bilirubin, albumin, and gamma-glutamyl transferase (GGT). Values above 1.5 times the upper limit of normal (ULN) were considered clinically relevantly elevated, as suggested in previous research (20). All blood samples were analyzed by Q^2^ Solutions, in the United Kingdom.

### 2.4 Statistics

Data are presented as median and interquartile range (IQR), unless otherwise specified. The Mann–Whitney U test was used to compare continuous outcomes between patients and controls, while Fisher’s exact test was applied for binary outcomes. For comparisons of continuous variables across multiple groups, we applied the Kruskal-Wallis test. Associations between two continuous variables were examined using Spearman’s rank correlation and linear regression. For the diagnostic accuracy testing, we used a linear regression model with interaction analysis, and sensitivity and specificity tests. Sensitivity analyses were performed, where relevant, to evaluate the impact of extreme outliers. *p*-values < 0.05 were considered statistically significant. All statistical analyses were performed using R version 4.3.0.

### 2.5 Ethics statement

This study was approved by The Danish National Committee on Health Research Ethics (H-22008166) and was conducted in accordance with the Declaration of Helsinki and Good Clinical Practice guidelines. Written informed consent was obtained from all participants.

## 3 Results

A total of 33 patients were included in the study. The median age at examination was 57 years (48–63), and the patients had been receiving HPS for a median duration of 10 years (5–17). Most patients (25/33) received lipid-containing HPS, and the majority (96%) were prescribed plant-based lipid emulsions containing soybean oil. Short bowel syndrome (SBS) was the most common cause of IF. Patient characteristics, including HPS composition, are summarized in Table 1. The patients were comparable to the controls regarding age, sex, and BMI.

**Table 1:** Patient characteristics.

Patient-related characteristics, median (IQR)	Patients (*N* = 33)	Controls (*N* = 14)	*P*-value
Sex, *N* (%)			1
Female	15 (45)	7 (50)	
Male	18 (55)	7 (50)	
Age at examination, years	57.0 (48.0–63.0)	57.5 (39.3–63.8)	0.82
Female	53.0 (47.5–65.0)	57.0 (36.5–58.0)	
Male	57.0 (49.5–61.8)	60.0 (50.5–65.5)	
Body mass index, kg/m^2^	22.6 (19.7–25.6)	24.0 (22.0–26.0)	0.15
Female	20.8 (18.6–26.0)	21.7 (21.1–23.5)	
Male	23.2 (20.3–25.0)	25.4 (24.8–27.8)	
Home parenteral support, median (IQR)			
Duration of HPS, years	10.4 (5.3–16.8)	-	
Total infusion volume, mL of HPS/day	2576.0 (2002.0–3627.0)	-	
Infusion duration, hours/day	11.0 (10.0–12.0)	-	
Frequency of HPS, days/week	7.0 (7.0–7.0)	-	
Composition of home parenteral support, median (IQR)			
HPS energy, kJ/day	6892.0 (4594.0–7893.0)	-	
Lipid infusion, *N* (%)	25 (76)	-	
Lipid type*, *N* (%)			
Fish oil based	0 (0)	-	
Plant based	24 (96)	-	
Mixed oil	1 (4)	-	
Lipids, g/day	14.3 (5.7.-22.9)	-	
Lipids, g/kg/day	0.2 (0.1–0.4)	-	
Lipids, kJ/kg/day	7.8 (4.4–17.5)	-	
Amino acids, g/day	73.0 (52.6–85.3)	-	
Amino acids, g/kg/day	1.1 (0.8–1.6)	-	
Amino acids, kJ/kg/day	24.9 (18.9–38.1)	-	
Glucose, g/day	314.3 (214.3–360.0)	-	
Glucose, g/kg/day	4.8 (2.9–6.2)	-	
Glucose, kJ/kg/day	84.0 (51.7–109.3)	-	
Cause of intestinal failure, *N* (%)			
Short bowel syndrome	26 (79)	-	
End-jejunostomy	22 (67)	-	
< 100 cm	8 (24)	-	
100–200 cm	6 (18)	-	
> 200 cm	8 (24)	-	
Jejuno-colonic anastomosis	4 (12)	-	
Jejuno-ileo-colonic-anastomosis	0 (0)	-	
Other	7 (21)	-	
Underlying disease leading to intestinal failure, *N* (%)			
Morbus Crohn’s disease	18 (55)	-	
Motility disorders	7 (21)	-	
Mesenteric ischemia	1 (3)	-	
Post-surgical conditions	7 (21)	-	

HPS, home parenteral support; kJ, kilojoule.

Statistics: Mann–Whitney U-test and Fisher’s exact test were used to test for differences between patients and controls.

*Plant oil-based emulsions contained soybean oil (Intralipid®, *n* = 3; Kabiven®, *n* = 17) or soybean and olive oils (Olimel®, *n* = 4). Mixed oil-based emulsions (SMOFlipid®, *n* = 1) contained soybean and olive oils, medium-chain-triglycerides, and fish oils. No purely fish oil-based emulsion were used in this cohort.

Three patients (9%) were excluded from LSM analyses due to technical failures during TE assessments. An additional 3 (9%) were excluded because the necessary equipment and specialized expertise required for the assessment were not available to our research team at the time of the study. Thus, a total of 27 patients were available for LSMs analyses. Two patients (6%) experienced claustrophobia and could not complete the MRI scan, leaving 31 patients for MRI-PDFF analyses. Blood samples were collected from all patients.

### 3.1 Liver stiffness

**Figure 1:**
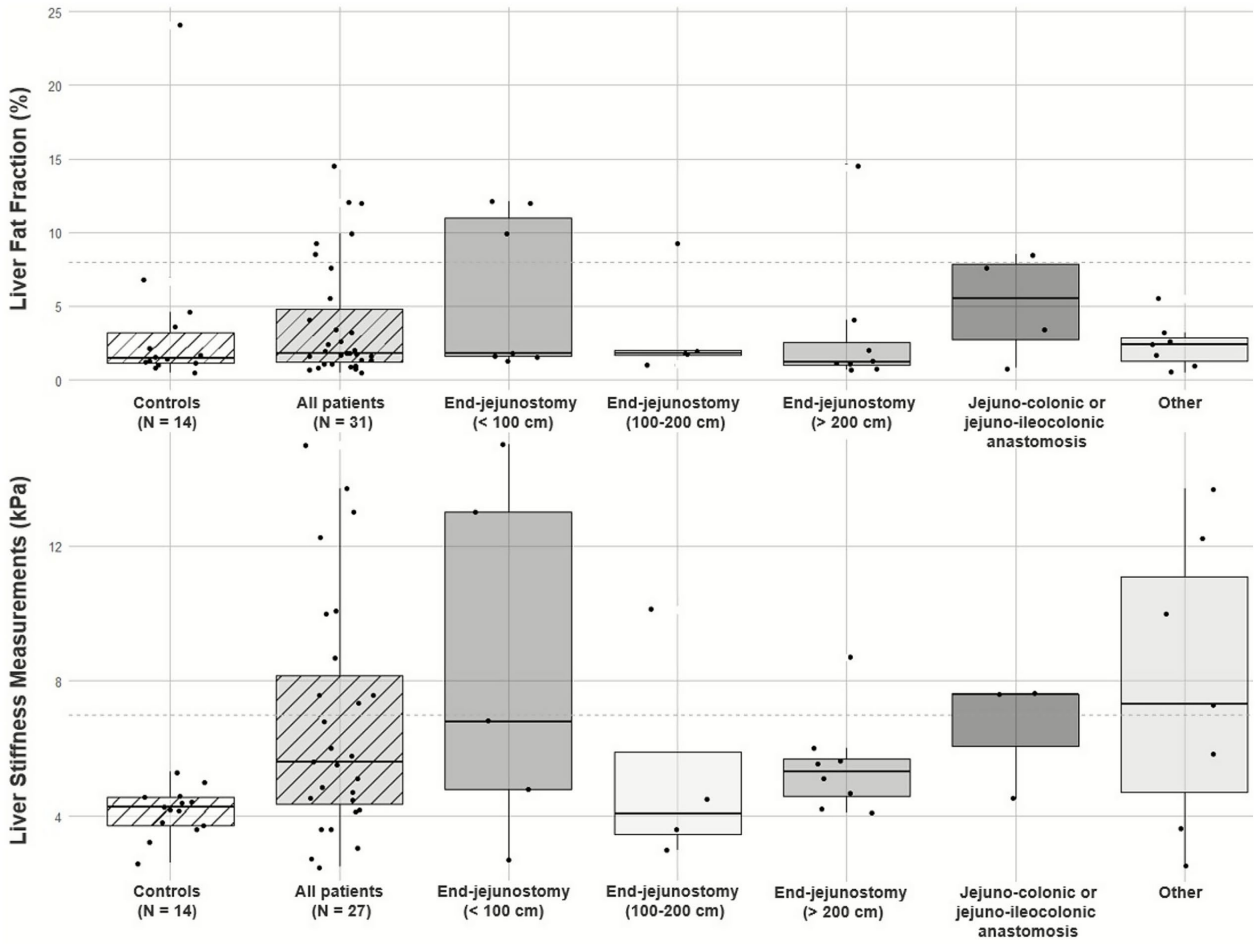
Liver Stiffness and Liver Fat Fraction in Patients and Controls. The hatched boxplots show the control group and the total patient group. The unhatched boxplots show the patient subgroups divided according to their different gastrointestinal anatomies. Box boundaries indicate the interquartile range, and the solid line shows the median. The dashed lines mark the cut-off values for liver stiffness (> 7 kPa) and liver fat fraction (> 8%). kPa, kilopascal.

The LSMs were significantly higher in patients compared to controls (5.6 kPa (4.4–8.2) vs. 4.3 kPa (3.7–4.6), respectively; *p* = 0.0079) (Figure 1). Additionally, a higher proportion of patients had elevated LSMs (> 7 kPa) compared to controls (37% vs. 0%; *p* = 0.0085). There was no significant difference in LSMs across the different bowel anatomy groups (*p* = 0.65), as shown in Figure 1. Patients who received HPS containing lipids had higher LSMs compared to those who did not receive lipids [6.8 kPa (5.0–10.1) vs. 4.4 kPa (3.8–4.9); *p* = 0.029]. Additionally, we found a positive correlation between LSMs and lipids per kilogram bodyweight (rho = 0.5; *p* = 0.0042), daily HPS energy content (rho = 0.4; *p* = 0.026), and energy per kilogram body weight (rho = 0.4; *p* = 0.022).

Linear regression analysis showed that each 1,000 kJ increase in daily HPS energy, was associated with a 0.6 kPa increase in LSMs (*β* = 0.6; 95% CI: −0.006 to 1.2; *p* = 0.052), although this association was only borderline significant.

Elevated LSMs were not significantly associated with estimated small bowel length, HPS duration, HPS daily volume, amino acids, or glucose per kilogram bodyweight.

### 3.2 Steatosis assessed by CAP and MRI-PDFF

The CAP values were 220 dB/m (184–253) in the patients and 224 dB/m (194–254) in the controls, with no significant difference between the groups (*p* = 0.59). Elevated CAP values (> 248 dB/m) were found in 30% (8/27) of patients, and 29% (4/14) of controls (*p* = 1). There was no association between CAP values and specific HPS components, HPS duration, HPS daily volume, HPS daily energy, or estimated bowel length.

The MRI-PDFF was 1.8% (1.2–4.8) in patients and 1.5% (1.1–3.2) in controls, with no significant difference between the groups (*p* = 0.43) (Figure 1). Elevated MRI-PDFF (> 8%) was observed in 19% (6/31) of the patients and 7% (1/14) of the controls (*p* = 0.41). MRI-PDFF was not associated with specific HPS components, HPS duration, HPS daily volume, HPS daily energy, or estimated bowel length.

### 3.3 Diagnostic accuracy tests

We explored the diagnostic accuracy of CAP by using MRI-PDFF as the reference standard. In the patient group, the sensitivity of CAP, referring to the proportion of true positive for steatosis, was 75% in the patients, while it was 100% in the controls. On the other hand, the specificity of CAP, indicating the detection of true negatives for steatosis, was similar in both groups, 82 and 77% in patients and controls, respectively.

**Figure 2:**
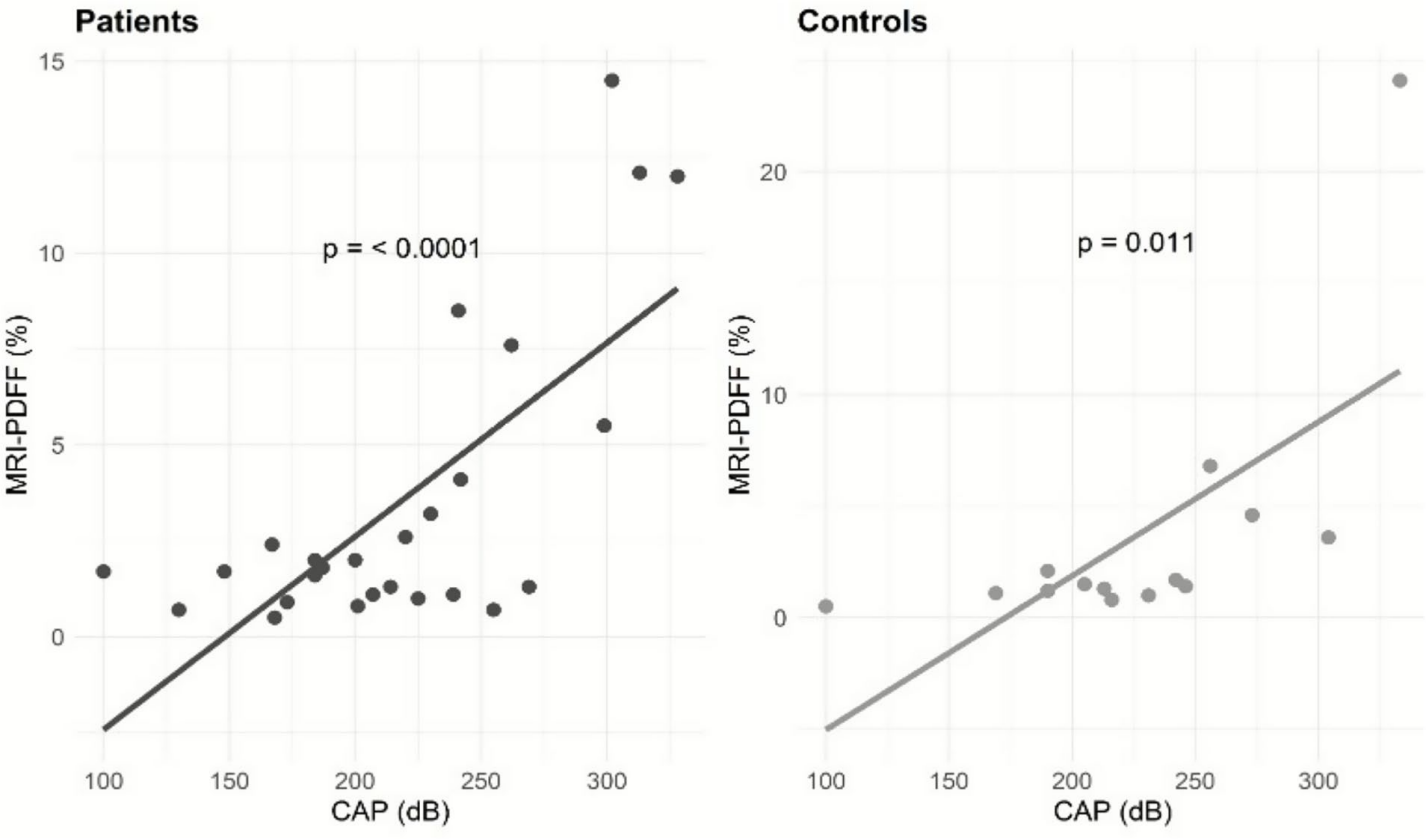
Relationship between MRI-PDFF and CAP measurements in patients and controls. Statistics: a simple linear regression analysis showed a significant increase in CAP as MRI-PDFF increased (for patients: *β* = 0.05; 95% CI: 0.03 to 0.07; *p* = < 0.0001 and for controls: *β* = 0.07; 95% CI: 0.02 to 0.1; *p* = 0.011). The relationship between MRI-PDFF and CAP did not significantly differ between the groups (*p* = 0.40). CAP, controlled attenuation parameter; dB, decibel; MRI-PDFF, magnetic resonance imaging-proton density fat fraction.

We observed a positive correlation between MRI-PDFF and CAP in both patients (rho = 0.5; *p* = 0.0037) and controls (rho = 0.7; *p* = 0.0033). A linear regression analysis showed that each 10 dB increase in CAP value, were associated with a 0.5% increase in MRI-PDFF for patients (*β* = 0.05; 95% CI: 0.03 to 0.07; *p* = < 0.0001) and a 0.7% increase in MRI-PDFF for controls (β = 0.07; 95% CI: 0.02 to 0.1; *p* = 0.011) (Figure 2). However, the relationship between MRI-PDFF and CAP did not significantly differ between the patient and control groups (*p* = 0.40) (Figure 2).

### 3.4 Laboratory parameters

**Figure 3:**
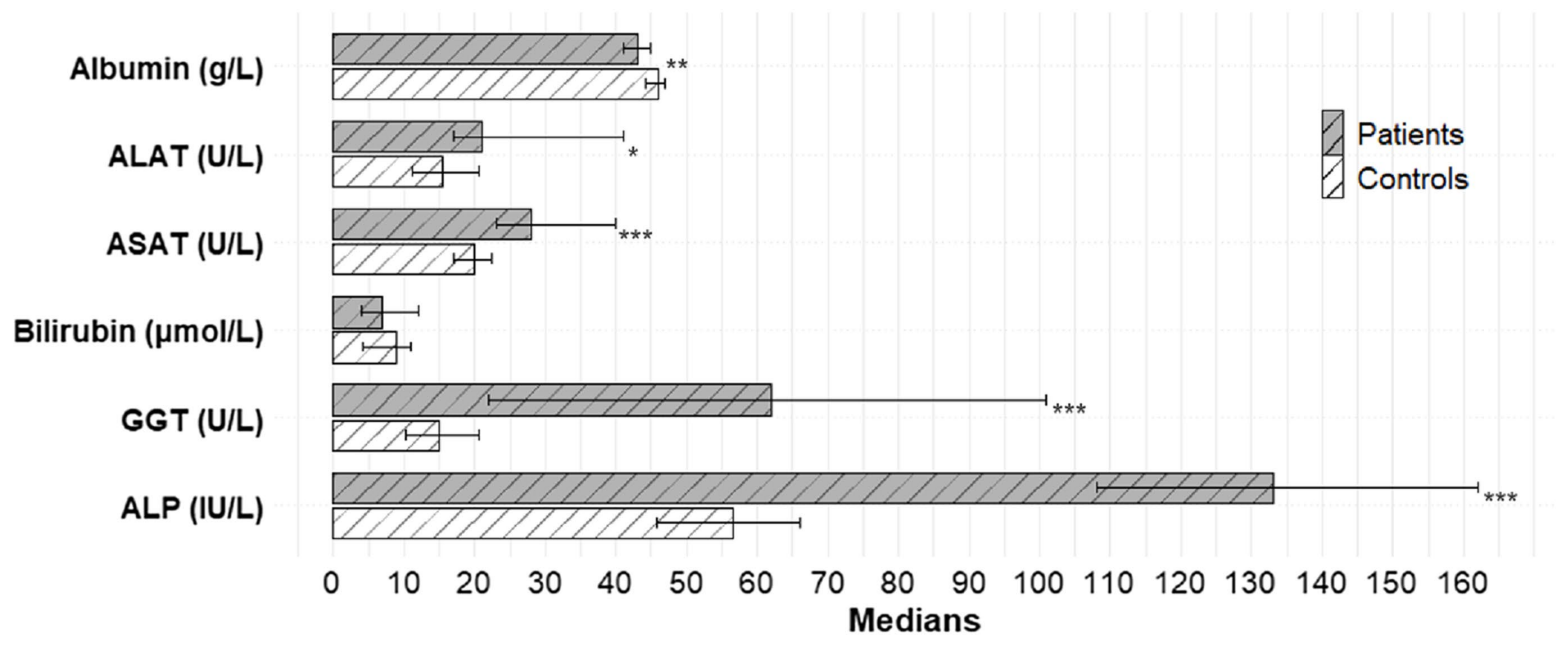
Distribution of liver function tests in patients and controls. The figure shows the median values of liver function tests in patients (grey bars) and controls (white bars). Statistics: a Mann–Whitney U-test was used to test for differences between patients and controls. *Indicates statistical significance: *p < 0.05; **p < 0.01; ***p < 0.001. ALP, alkaline phosphatase; GGT, gamma-glutamyl transferase; ASAT, aspartate aminotransferase; ALAT, alanine aminotransferase.

In the patient group, ALP, GGT, ALAT, and ASAT levels were significantly higher compared to the control group (133 IU/L vs. 57 IU/L; *p* = < 0.0001, 62 U/L vs. 15 U/L; *p* = <0.001, 21 U/L vs. 16 U/L; *p* = 0.032, and 28 U/L vs. 20 U/L; *p* = <0.001, respectively). Albumin was lower in patients compared to controls (43 g/L in patients vs. 46 g/L in controls; *p* = 0.0028). Total bilirubin levels were similar in patients and controls (7 μmol/L vs. 9 μmol/L; *p* = 0.93), and none of the patients had elevated bilirubin levels (Figure 3). ALP and GGT levels > 1.5x the ULN were observed in 21 and 45% of patients, respectively.

## 4 Discussion

This study presents novel and potentially clinically relevant insights into liver abnormalities in a subset of adult patients with IF receiving long-term HPS. We observed a varied presentation of liver manifestations, with elevated liver stiffness, as assessed by TE, appearing more prevalent than steatosis, as measured by CAP and MRI-PDFF. A cholestatic pattern in liver enzymes was also demonstrated. We identified HPS energy and lipid content as potential risk factors for elevated liver stiffness.

Our study showed elevated LSMs in 37% of patients. In contrast, Knop et al. (30) report a prevalence of 18% (4/22) of significant or advanced fibrosis or cirrhosis (> 7 kPa) measured by TE, without significant dynamic changes within a 12-month follow-up period. The difference may be due to the longer HPS duration in our cohort (median 10 years in our study vs. 30 months in Knop’s study), suggesting that liver manifestations may progress over time. While TE has been a reliable surrogate marker for fibrosis in other liver diseases, it should be interpreted cautiously in patients with IF since no significant correlation has been reported between TE and the stages of histologic fibrosis (31, 32). Moreover, factors such as inflammation (25, 33, 34), cholestasis (25, 35), diet (23, 24), and altered portal blood flow (25, 36), as well as disruption of the enterohepatic bile acid circulation and synthesis due to heterogeneous intestinal anatomies, may influence the results.

In our cohort, patients receiving lipids appeared to have higher LSMs. It is important to note that patients receiving lipids often have poorer intestinal function, reduced portal blood flow due to shorter remnant bowel lengths, and typically require a higher HPS energy load. However, different bowel anatomy did not appear to affect LSMs across the groups (Figure 1) which could be due to the small number of patients within each anatomy group. Patients with IF often have disruption of the enterohepatic bile acid circulation, low fibroblast growth factor 19 levels due to ileal resection, and high 7α-hydroxy-4-cholesten-3-one levels, indicative of accelerated bile acid production to compensate for fecal losses (9, 16, 37–40). Therefore, it may not solely be the amount or composition of HPS, but rather a synergy of factors that could contribute to development of liver fibrosis. In addition, the type of lipid emulsion used in HPS may also affect liver outcomes, as different lipid types, particularly soybean-based emulsions, have been associated with liver dysfunction (9, 12, 19). Among the patients receiving lipid emulsions in this cohort, nearly all (96%) received lipids containing soybean-oil, which limited the possibility of further analyses across lipid types.

In this study, we opted to assess patients under their habitual conditions. The patients adhered to their prescribed HPS to maintain their usual hydration status, as altered hydration status theoretically may affect venous pressure and thereby influence TE results (23–25, 36). Interestingly, the LSMs results in this study closely aligned with those of a previous study conducted at our IF center under oral fasting conditions, indicating that oral fasting may have had a limited impact on LSMs results in patients with IF (41). Future studies should address these factors to better decide the optimal timepoint and conditions related to HPS infusions, oral intake, and other factors to optimize standardized conditions related to the performance of TE in patients with IF.

In contrast to existing literature, we did not observe a high degree of steatosis in our cohort of patients (13, 42–44). However, another study conducted at our IF center, which only included SBS-IF patients, reported a steatosis prevalence of 65% as measured by CAP (41). Differences in steatosis prevalence could potentially be explained by variations in the selection of patient populations based on in- and exclusion criteria, differences in HPS duration, HPS volume, BMI, mixed underlying diagnosis, bowel anatomy and fasting regimens on the study day. This underscores the potential importance of evaluating similar IF patient groups when comparing IFALD manifestations across studies. In this study, one participant in the control group exhibited a markedly elevated MRI-PDFF measurement (Figure 2). To assess the influence of this outlier on the data analyses, we conducted a sensitivity analysis excluding this individual. The statistical significance of the findings was unaffected, indicating that the outlier did not affect the overall results.

The relatively low prevalence of steatosis in our study population may be explained by the amount of glucose and type of HPS lipids provided to these patients, since these can vary significantly among IF centers across the world depending on their HPS regimens and potentially on the ability to “tailor-make” an optimally individualized HPS program.

To our knowledge, this is the first study to examine the correlation between MRI-PDFF and CAP in patients with IF and controls. Studies in patients with non-alcoholic fatty liver disease have reported Spearman’s rho correlation coefficients of approximately 0.5 (45, 46). These correlations are similar to what we observed in our patient group. However, the correlation observed in the control group was notably stronger (rho = 0.7), suggesting that there could be a more consistent relationship between CAP and MRI-PDFF in controls. When examining our linear regression models, we did not find a significant difference in the relationship between CAP and MRI-PDFF between the patients and the controls (Figure 2). This may suggest that the relationship between the groups is similar. However, it is also possible that the small sample size limits the statistical power to detect a difference across the groups. In line with this, CAP appeared to have higher sensitivity in controls compared to patients (100% vs. 80%), indicating that CAP may be more effective at identifying steatosis in healthy individuals compared to adult patients with IF. We hypothesize that the heterogeneity of patients with IF and the complexity and multiplicity in the pathophysiological contributors of IFALD may complicate the assessment of hepatic steatosis in this patient group.

Our finding of 21 and 45% of patients with elevated (>1.5x ULN) ALP and GGT levels, respectively, demonstrate a cholestatic pattern in the blood samples in this patient population. These findings are consistent with those reported by Frezet et al., who reported biochemical cholestasis (ALP and/or GGT > 1.5x ULN) in 21% of patients with IF receiving HPS (43). Due to the cross-sectional study design, we report only a single assessment of the liver biochemistry. However, other longitudinal studies have demonstrated elevated ALP and GGT levels in patients monitored over longer periods, suggesting ongoing liver injury over time (13, 20).

The strengths of this study include the relatively large group of patients with IF with a notably long HPS duration (median 10 years (5–17)), the inclusion of a healthy control group, and the novel use of non-invasive tests in such patients, especially the validated and accurate MRI-PDFF method (28, 29). However, MRI-PDFF also has some practical limitations, as it is less accessible, cannot be used in patients with MRI contraindications, and quantifies liver fat only (47). A limitation of this study is that, although the patient group is relatively large within the field of IF research, where study populations are generally small, the rarity of the disease does still preclude large sample sizes, as is the case in this study. Thus, the small absolute number of patients carries a risk of insufficient statistical power and may limit the generalizability of the findings to broader IF populations. Furthermore, this was a single-center study including a specific group of patients with IF, and differences in underlying diseases, treatments, and HPS regimens in other IF centers should be considered when interpreting the results. In addition, potential selection bias from the eligibility criteria may also affect generalizability. To address this limitation, future international multicentre studies with larger study populations are warranted. Lastly, the limitations of a cross-sectional design prevents this study from drawing conclusions regarding causality and therefore the findings are reported as associations rather than casual relationships.

## 5 Conclusion

In this study of a well-defined cohort of adult patients with IF receiving long-term HPS, we observed a mixed presentation of liver manifestations, characterized by a cholestatic pattern in liver enzymes, and elevated LSMs appearing more prevalent than steatosis. Our data suggest that HPS energy and lipid content, indicative of poor intestinal function and severe IF, may be potential risk factors for developing increased liver stiffness.

Overall, with a growing population of individuals with long-term IF, and IFALD now being one of the most serious IF complications, it is crucial to gain a better understanding of the causes and dynamics of this complex condition. A first step could be a more systematic approach to identifying patients at highest risk of developing clinically significant IFALD. This could be followed by larger prospective longitudinal studies using state-of-the-art non-invasive methods and biomarkers to evaluate the progression of fibrosis and steatosis over time in this subset of patients with IF. As new drugs for treating steatosis and fibrosis become available, these - or more specific drugs designed for patients with IF- would also be welcome in establishing their efficacy and safety in the rare condition of progressive IFALD.

## Data Availability

The raw data supporting the conclusions of this article will be made available by the authors, without undue reservation.
